# Revisiting the conceptualization of social sustainability from a health promotion perspective: a scoping review

**DOI:** 10.1177/14034948241277863

**Published:** 2024-09-26

**Authors:** Ragnhild Martinsen Ånestad, Emma Charlott Andersson Nordbø, Camilla Ihlebæk

**Affiliations:** 1 Department of Public Health Science, Norwegian University of Life Sciences, Norway; 2 The Center for Evidence-Based Public Health, Norway; 3 Faculty of Health and Social Work Studies, Østfold University College, Norway

**Keywords:** Social sustainability, health promotion, socio-ecological model, neighborhood, city, local community

## Abstract

**Aims:**

Both social sustainability and health promotion emphasize the development of local communities that satisfy human needs and enhance health and well-being. This scoping review aimed to identify frameworks, components, and operationalizations of social sustainability described in peer-reviewed articles and systematize the results from a health promotion perspective.

**Methods:**

Six databases were searched for relevant articles following the JBI methodology and PRISMA guidelines. Articles were included if they provided a unique framework for social sustainability, that is, a conceptual model outlining the essential components of this concept. Information concerning the components of social sustainability and their operationalizations was analyzed through a six-step narrative synthesis. The final step involved categorizing the operationalizations of social sustainability into distinct levels by employing a socio-ecological model as an analytical tool.

**Results:**

This review identified 22 articles presenting a social sustainability framework. The frameworks covered 11 components, of which social equity, safety, and neighborhood quality were the most frequent, while only five included health and well-being. The frameworks commonly provided practical interpretations of the concept with limited theoretical considerations. Furthermore, the identified operationalizations revealed a diverse understanding of social sustainability, encompassing all levels of the socio-ecological model applied.

**Conclusions:**

**Health promotion research can offer theoretical and empirical insights that enhance the understanding of social sustainability, especially how societal, physical, and social determinants of health and well-being interact to create socially sustainable places. Likewise, the social sustainability literature may provide valuable knowledge underscoring the importance of contextual factors of health and well-being within the field of health promotion.**

## Background

Social sustainability is recognized as a crucial component of sustainability [1]. Yet, compared to the economic and environmental dimensions of sustainability, the social dimension has received less attention [2] and has even been referred to as the forgotten pillar [3]. Nevertheless, governments and policymakers are increasingly aware of the importance of integrating the social aspects into sustainable development [4, 5], reflected by the expanded volume of research on social sustainability over the last couple of years [6].

Scholars across various fields have studied social sustainability, leading to the development of multiple frameworks aimed at defining and conceptualizing this concept [7–9]. In this study, a framework refers to a conceptual model outlining the main components of the studied concept. Social sustainability is context-dependent [7], and some frameworks have been developed explicitly for certain spatial levels, such as neighborhoods [8, 10] or cities [11], or a given geographical area, such as India [12, 13] or China [14, 15]. The frameworks for social sustainability vary in complexity and terminology, posing challenges when comparing studies. Some researchers have even concluded that the concept appears fragmented, over-simplified, and under-theorized [2, 16–18].

Earlier reviews have identified several definitions of social sustainability [2, 19–22]. For example, Woodcraft [9] defines social sustainability as a process for “creating sustainable, successful places that promote well-being by understanding what people need from the places in which they live and work.” Larimian and Sadeghi [23] focus on conditions by defining a socially sustainable neighborhood as a place that “provides residents with equitable access to facilities, services, and affordable housing; creates a viable and safe environment for interaction and participation in community activities; and promotes a sense of satisfaction and pride in the neighborhood in a way that people would like to live there now and in the future.” McKenzie [24] combines both perspectives, suggesting, “Social sustainability is a positive condition within communities and a process within communities that can achieve that condition.” Although definitions vary, many agree that social sustainability is concerned with creating societal, physical, and social environments that satisfy human needs and ensure the well-being of present and future generations within the places they live [3, 8, 9, 16].

This understanding of social sustainability aligns well with the widespread consensus within health promotion that the communities where people live their everyday lives represent important arenas for developing and sustaining good health and well-being [25, 26]. Modern definitions of health state that good health and well-being are resources for everyday life, emphasizing social and personal assets and physical capabilities [25, 27]. According to this broad definition, health promotion involves “the process of enabling people to increase control over and to improve their health” [25, 28], and it encompasses actions aimed at modifying social and environmental conditions to support both individual and collective health [28]. Health promotion is an all-encompassing social and political process [28], necessitating collaboration across multiple sectors and stakeholders [25]. Calls for multidisciplinary collaborations have also emerged in social sustainability literature [17, 29]. Hence, there is considerable overlap between social sustainability and health promotion [30]. Interestingly, the concept of social sustainability generally appears to be absent from the research literature on health promotion. Likewise, knowledge and theories from the field of health promotion seem to be overlooked in the social sustainability literature. This is surprising given the substantial overlap between the two fields and their shared emphasis on the societal, physical, and social factors that create local communities where people can thrive and that satisfy human needs, health, and well-being [30].

Several social sustainability studies have investigated the impact of urban forms (e.g., density, building typology, mixed land use) on social outcomes (e.g., social equity, safety, social interaction) [12, 31-34], or they have assessed the level of social sustainability within different spatial units [10, 11, 13, 35]. These studies have applied a variety of measurement variables, frequently based on political goals, expert knowledge, or the background or professional field of the researchers [11–13]. Such an extensive range of operationalizations demands a systematic approach to the literature to raise awareness within research, practice, and policy across the different fields involved in social sustainability. The field of health promotion has a long tradition of applying a socio-ecological perspective to health and well-being [36, 37]. This perspective emphasizes that factors in the environment, such as social relationships, physical features, and legislation, alongside individual factors like gender, age, biology, and lifestyle, collectively influence people’s health and well-being. Several models, like “The Health Map for the Local Human Habitat” [38] and “The Rainbow Model” [39], have been developed to systematize and visualize these social determinants of health [38, 39]. Socio-ecological models are not causal; however, they do offer simplified visualizations of the complexity of factors affecting people’s health and well-being. Adopting a socio-ecological model as an analytical tool could therefore provide a solid ground for systematizing the conceptualizations and operationalizations of social sustainability. Furthermore, by applying a socio-ecological model, the social sustainability literature may become more accessible to scholars within the field of health promotion who have not yet engaged in social sustainability research.

Although attempts have been made to review the existing literature on social sustainability, several reviews lack methodological rigor and transparency in their approach [18, 21, 40, 41]. In addition, systematic reviews have been restricted to the built environment or the physical–material community context [6, 17], housing [19], or urban disciplines [20]. To our knowledge, a systematic review has not yet been conducted from a health promotion perspective. Therefore, we aimed to conduct a scoping review to identify frameworks, components, and operationalizations of social sustainability within cities, local communities, and neighborhoods. Furthermore, we aimed to provide new insight into the conceptualization and operationalization of social sustainability by synthesizing the results from a health promotion perspective using a socio-ecological model as an analytical tool.

## Methods

A scoping review is a type of systematic review appropriate when the objective is to clarify key concepts or identify characteristics or factors related to a concept [42, 43]. Therefore, a scoping review was conducted based on existing methodological guidelines [43, 44]. The review procedure included establishing the eligibility criteria, developing the search strategy, selecting sources of evidence, extracting data, and synthesizing data. Each of these steps will be described in the following sections. We adhered to the PRISMA-ScR statement for the reporting of the scoping review [45].

## Eligibility criteria

Articles were eligible for inclusion if they addressed the social dimension of sustainability. This means that articles investigating sustainability in general, without paying specific attention to the social dimension, were excluded. To be included, the publications were required to propose a unique framework for social sustainability. A framework herein refers to a conceptual model presenting the main components of the concept of interest. Furthermore, the contexts of interest in this study were settings for human settlements such as cities, neighborhoods, and local communities. Studies presenting a framework for small-scale housing development or urban regeneration projects were omitted. All types of quantitative and qualitative primary studies and theoretical papers were eligible for inclusion. Literature reviews and other evidence syntheses were also considered, but these papers were only included if the authors provided a unique framework for social sustainability. The publications had to be written in English and published in a peer-reviewed journal. Commentaries, editorials, and gray literature were not considered for inclusion.

## Search strategy and information sources

To identify peer-reviewed publications, searches were undertaken in the following databases: Ovid MEDLINE(R) ALL, Scopus, EMBASE (OVID), PsycINFO (OVID), Web of Science, and CINAHL (EBSCOhost). We utilized a three-step search strategy as recommended by Aromataris and Munn [46]. First, preliminary searches were conducted in all six databases to identify index terms and keywords for social sustainability (the concept of interest) and for city, neighborhood, and local community (the contexts of interest). These initial searches informed the development of a complete search strategy tailored to each database. Subsequently, comprehensive searches were performed in all the databases without imposing any restrictions on the year of publication. The entire search was initially carried out in March 2021, followed by an updated search in June 2022. The search strategy applied in Ovid MEDLINE(R) ALL is detailed in Supplemental material 1. Finally, the reference lists of all included articles were screened to identify any additional relevant publications.

## Selection of sources of evidence

All identified records were uploaded into EndNote 20™ (Clarivate Analytics, PA, USA), and duplicates were removed. The remaining references were transferred to Rayyan [47], where two pairs of reviewers independently screened the titles and abstracts. RMÅ and ECAN screened 50% of the records, whereas the other pair, RMÅ and CI, screened the other half. The same pairs of reviewers separately examined all records selected for full-text retrieval for eligibility based on the predefined inclusion criteria. Disagreements between the reviewers regarding whether to include or exclude a paper were resolved through consensus in group discussions among all reviewers. An overview of all included articles is presented in Supplemental material 2. Supplemental material 3 provides an overview of all articles excluded based on full-text assessment and the reasons for their exclusion.

## Data extraction

A data extraction form was developed in the Microsoft Access database to record information from each included article systematically. Before data extraction, all reviewers piloted the form, and adjustments were made based on group discussions. The following general information was extracted from each article: publication information (i.e., title, author, year of publication, journal, and geographic origin) and study characteristics (i.e., type of study, objectives, methods, and context). Moreover, data concerning the frameworks were extracted. This encompassed information about the framework’s key characteristics and components and details regarding framework development. The publications described the components using various terms, such as dimensions, principles, criteria, and factors. For consistent terminology, we refer to them as components in this scoping review. In addition, information detailing the operationalizations of each component was extracted, if provided. An operationalization herein includes measurement variables applied or suggested for social sustainability and proposed policy initiatives. The data extraction form utilized for recording the information is provided in Supplemental material 4.

## Data synthesis

The data synthesis started by categorizing all included studies based on their general characteristics (geographic origin, context of interest, and year of publication). This was followed by a narrative synthesis, a process for synthesizing information from studies through text analysis [48]. The narrative synthesis progressed through six stages. In the first stage, we classified the frameworks according to the purpose behind their development (i.e., “assessment” or “policy and planning”). The second stage involved categorizing the types of literature reviews conducted to support the frameworks (i.e., systematic, unsystematic, and not described). In the third stage, strategies and methods utilized in the included papers for identifying the main components of social sustainability were sorted into six non-mutually exclusive categories (i.e., focus group interviews, consensus/workshop sessions, thematic analysis, existing frameworks, questionnaires/indicators, and not described). The inclusion of measurement variables for social sustainability assessment was registered (i.e., yes/no) in the fourth stage. The fifth stage included categorizing the components of social sustainability based on the terminology and descriptions provided in the articles. Finally, an analysis of the operationalization of each identified component was conducted in the sixth stage. Inspired by socio-ecological models commonly applied in the field of health promotion, such as the Health Map model [38] and the Dahlgren–Whitehead rainbow model [39], we identified five levels of operationalizations: population (e.g., age, gender), social environment (e.g., perceived safety, social relationships), physical environment (e.g., access to services and facilities), societal structures and legislation (e.g., distribution of resources), and global and ecological environment (e.g., reducing consumption level). For example, if a component of social sustainability was operationalized by measuring the availability or accessibility of services and facilities in the neighborhood, we categorized this operationalization as an aspect of the “physical environment.” Conversely, if a component was operationalized by measuring the level of social interactions between neighbors, we categorized this operationalization as an aspect of the “social environment.”

## Results

**Figure 1.**
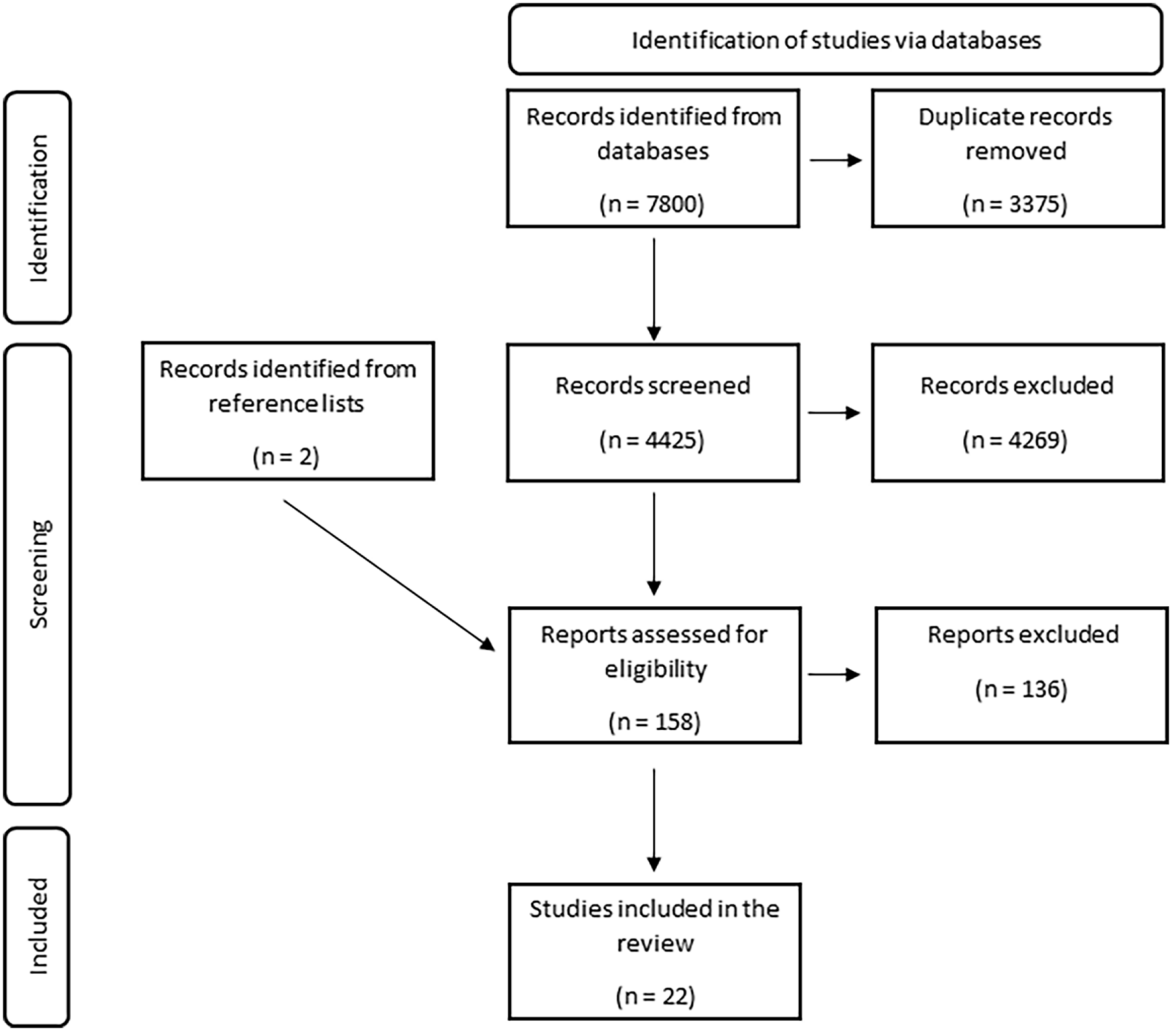
A modified PRISMA flow chart of the search and review process [49].

Through the comprehensive search, we identified 7800 references (Figure 1). After removing duplicates, 4427 titles and abstracts were screened against the established inclusion and exclusion criteria. A total of 158 articles were retrieved for full-text review, of which 22 met the inclusion criteria (Figure 1).

The majority of the studies were published between 2015 and 2022 (Table I). Most studies presented a framework for social sustainability in neighborhoods (*n* = 8) or cities (*n* = 8), and there was a preponderance of frameworks developed within an Asian context (*n* = 10). Furthermore, most of the studies were published within research areas such as architecture (*n* = 12) or urban planning (*n* = 5) (Supplemental material 2).

**Table I.** Descriptive characteristics of the studies included in this review.

Characteristic	Category	Count	Reference
Geographic origin	Asia	10	10–15, 31, 32, 50, 51
	Oceania	4	23, 29, 52, 53
	Europe	2	7, 54
	Africa	1	35
	North America	1	3
	Not specified	4	8, 55–57
Context of interest	Neighborhood	8	7, 8, 10, 23, 31, 32, 50, 51
	City	8	3, 11–13, 35, 52–54
	Community	1	15
	Region	1	29
	Multiple levels	1	14
	Not specified	3	55–57
Year of publication	1993–2009	1	50
	2010–2014	6	7, 10, 15, 29, 52, 57
	2015–2019	11	3, 8, 11–14, 23, 31, 35, 54, 56
	2020–2022	4	32, 50, 51, 55

The narrative synthesis revealed that 15 frameworks were developed for assessing social sustainability, whereas seven provided directions for policy and planning (Table II). In most cases, the authors conducted an unsystematic literature search to support the development of the framework (*n* = 18). We found nine articles in which the components of social sustainability were identified and incorporated into the frameworks based on existing questionnaires or indicators, whereas in six papers, consensus concerning which components to include was reached through consensus/workshop sessions. We identified 11 components of social sustainability, of which social equity, safety, and neighborhood quality recurred the most (Table II). The most common components are presented below.

**Table II.** Key characteristics of frameworks for social sustainability.

Characteristics of frameworks	Category	Count	Reference
Aims	Assessment	15	3, 7, 8, 10–13, 23, 31, 32, 35, 50–52, 54
	Policy and planning	7	14, 15, 29, 53, 55–57
Literature review	Systematic	1	8
	Unsystematic	18	3, 10–14, 23, 29, 31, 32, 35, 50–52, 54–57
	Not described	3	7, 15, 53
Strategies for identification of main components	Focus group interviews	1	10
	Consensus/workshop sessions	6	11–13, 23, 29, 32
	Thematic analysis	4	8, 13, 23, 56
	Existing frameworks	4	7, 35, 52, 54
	Questionnaires/indicators	9	3, 8, 10, 12, 13, 23, 50, 51, 57
	Not described	6	14, 15, 31, 53, 55, 57
Measurement variables included	Yes	12	3, 8, 10–13, 23, 31, 32, 50, 51, 54
	No	10	7, 14, 15, 29, 35, 52, 53, 55–57
Main components of social sustainability	Social equity	17	3, 7, 8, 12–15, 23, 29, 31, 32, 51–57
	Safety	12	7, 8, 10, 13, 23, 31, 32, 35, 50, 51, 55, 56
	Neighborhood quality	11	8, 10, 11, 13, 23, 31, 32, 50, 53, 54, 56
	Participation	10	7, 8, 10, 11, 15, 23, 29, 50, 51, 57
	Place attachment	9	7, 8, 11, 23, 31, 35, 50, 51, 53
	Social capital	9	3, 7, 8, 10–12, 15, 23, 29, 31, 32, 35, 50–52, 55, 57
	Social interaction	9	7, 8, 10, 11, 15, 23, 31, 50, 51
	Demographics	5	7, 8, 13, 31, 50
	Health and well-being	5	3, 10, 13, 14, 51
	Environmental responsibility	3	50, 56, 57
	Basic needs	3	3, 15, 52

**Figure 2.**
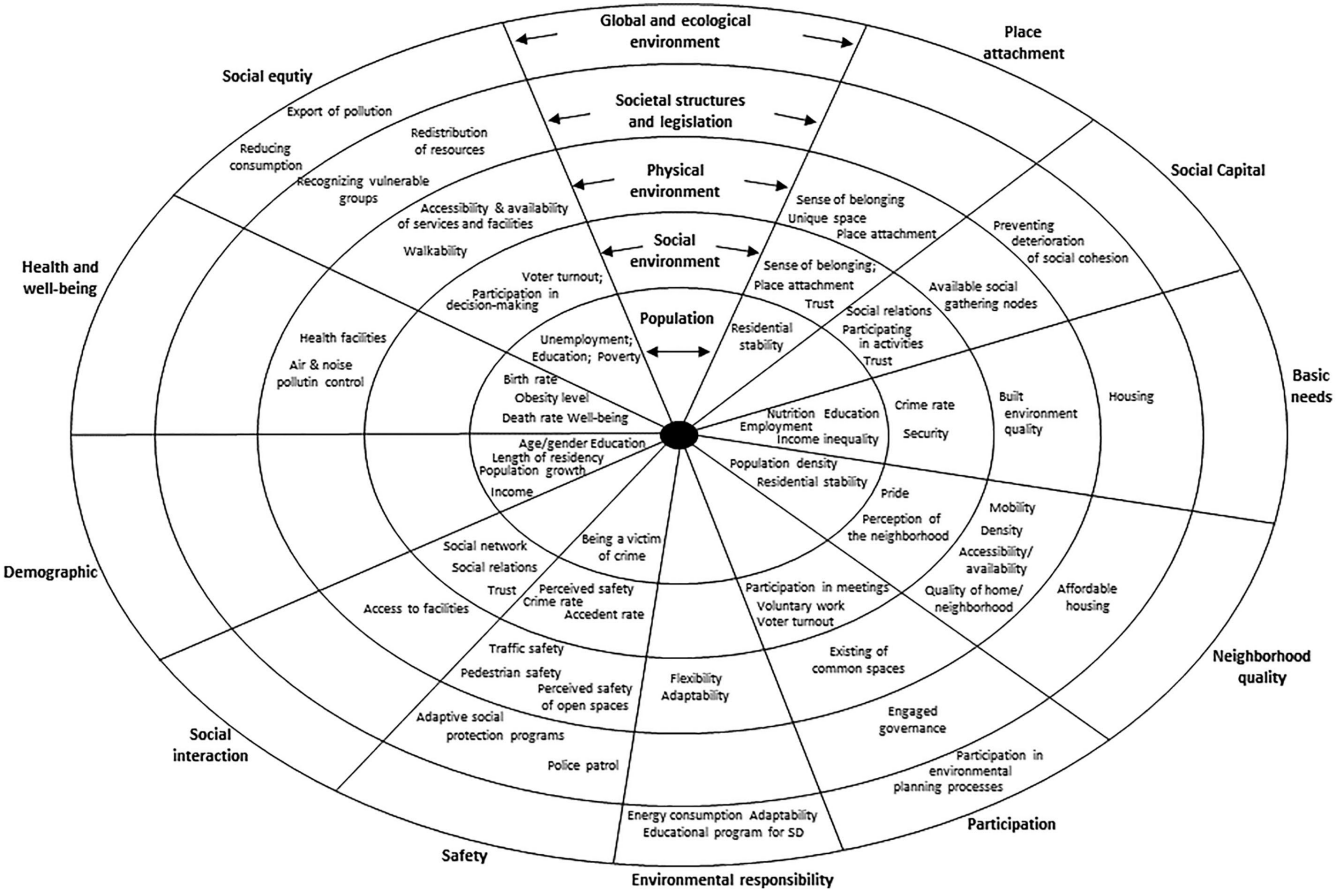
The socio-ecological model illustrating components and operationalizations of social sustainability.

Social equity was included in 17 frameworks (Table II), and this component was commonly described as equal access to services and facilities, equal distribution of resources, or equal opportunities to participate in decision-making processes regardless of sociodemographic or socioeconomic differences. The analysis showed that the operationalizations of social equity covered all levels in the socio-ecological model (Figure 2). Most frequently, though, social equity was operationalized as different aspects of the physical environment. This included measurements of perceived access to services and facilities [12, 23, 31, 51], the mere presence of services and facilities [3, 8], or distance to services and facilities [8, 54]. However, some studies operationalized social equity in terms of societal structures and legislation, for example, as policy initiatives targeting the redistribution of resources [55, 56], as aspects of the social environment, such as participating in decision-making processes [29] or voter turnout [3], or as an aspect of the population, for example, by assessing the unemployment rate [3], the school enrollment rate [13], or the poverty level [13].

In total, 12 frameworks included safety as a component, most frequently operationalized as aspects of the physical and social environment (Figure 2). The presence of well-connected pedestrian and cycling networks [10, 32], traffic safety [8, 32, 51], or the perceived safety of open spaces [8] were examples of operationalizations categorized as the physical environment, while perceived safety in the neighborhood [7, 8, 23, 31] and the crime rate [13] were examples of operationalizations grouped as the social environment.

Neighborhood quality was identified as a component of social sustainability in 11 frameworks (Table II). The operationalizations of this component represented four levels in the socio-ecological model. However, most commonly, neighborhood quality was assessed by objective measurements of the physical environment, such as the mobility options [10, 32] and urban density [8, 32, 56], or by subjective measurements of the perceived accessibility, availability, or quality of services and facilities [10, 11, 50, 54]. Some also operationalized neighborhood quality as perceptions of the neighborhood as a place to live, which was categorized as aspects of the social environment [23, 31, 32] (Figure 2).

The results showed that participation, social interaction, and social capital were frequently included as components in frameworks for social sustainability (Table II). However, we found inconsistencies in the descriptions and inclusion of these three components across studies. In several frameworks, participation and social interaction were presented as individual components of social sustainability [7, 8, 10, 15, 23, 50]. In contrast, some frameworks included participation and social capital as individual components [21, 54], and one included all three components [11]. Participation referred to involvement in community activities and decision-making processes, and this component was commonly operationalized by measuring aspects of the social environment, for example, the interest in or frequency of participating in decision-making processes [10, 11, 50] or involvement in voluntary work [10, 23]. Participation was also operationalized by assessing features of the physical environment, such as the existence of common spaces [51], societal structures and legislation aspects, such as engaged governance [29], and the global and ecological environment, including policy initiatives aimed at broadening participation in environmental planning processes [57] (Figure 2). Social interaction covered the relationship between inhabitants, and this component was operationalized as the level of contact between residents [8, 50] or the size of social networks [8, 23, 50], which encompass aspects of the social environment (Figure 2). Some studies operationalized social interaction by measuring physical environment features, such as the accessibility of facilities and services for activities [10, 51]. Social capital was commonly operationalized as social interactions [11, 12, 52], participation in activities [12, 32, 52], and trust [11, 12], which represent the social environment (Figure 2). Some also operationalized social capital as an objective assessment of the physical environment, for example, the availability of social gathering nodes, distinctive urban design, and high-quality services and facilities [32].

In total, nine frameworks included place attachment as a component of social sustainability (Table II), and this component was described as emotional bonds between people and places. The operationalization of place attachment targeted three levels in the socio-ecological model: physical environment, social environment, and population level. Examples of operationalizations were assessments of a sense of belonging to the place [8, 11, 23, 51] (both the physical and social environment), a sense of belonging to the community [11, 23, 50], and pride in the neighborhood [8, 31] (social environment), as well as residential stability [8, 11, 23] (population level) (Figure 2).

Only five frameworks included health and well-being as a component of social sustainability (Table II). Health and well-being was operationalized either as aspects of the physical environment (e.g., the presence of health facilities [10] and air pollution control [51]) or by using objective measures of health in the population (e.g., obesity level [3]) (Figure 2).

## Discussion

The narrative synthesis revealed 15 frameworks for assessing social sustainability and seven frameworks developed for policy and planning purposes. Most frameworks have been developed within an Asian context, while only two have considered social sustainability within a European context. Furthermore, most studies have been published in research areas such as architecture and urban planning. The frameworks commonly offered a practical interpretation of the concept with few theoretical considerations, and were developed based on unsystematic literature reviews. The conceptualizations of social sustainability within these frameworks encompassed 11 components, of which social equity, safety, and neighborhood quality were most prevalent. However, authors operationalized social sustainability components in diverse ways across all levels of the socio-ecological model. Some components, like social equity and neighborhood quality, were predominantly operationalized as aspects of the physical environment. Interestingly, only five frameworks included health and well-being, and this component was either operationalized as features of the physical environment or through objective measures of health in the population.

As in our study, previous reviews have found that the literature on social sustainability has been centered around key components [6, 17, 58], implying that the conceptualization of social sustainability may be less chaotic than previously indicated [18]. Nonetheless, our results suggest that despite a growing interest in investigating social sustainability over the last two decades, the concept has a vague theoretical foundation, as indicated by earlier studies [2, 16, 17]. The reviewed studies provided little theoretical consideration concerning the components or causal interactions between them. Furthermore, parts of the literature exhibit confusion concerning theoretical understanding, as exemplified by the concept of social capital. Although social capital has several theoretical understandings, this concept is generally recognized as consisting of certain key dimensions. According to Putnam [59, 60], central dimensions of social capital are social networks and participation in the community through voluntary organizations or other civic engagement. Another critical dimension of social capital is generalized trust [61]. Our results showed that although some studies adhered to this understanding [3, 7, 8, 11, 29, 32, 35], the conceptualizations of social capital varied. In some frameworks, social capital and participation were included as separate components [29, 57], several frameworks included both social interaction and participation without specifically referring to social capital [10, 15, 50], and one framework included social capital, participation, and social interaction as separate components in their conceptualization of social sustainability [11]. This diversified interpretation highlights the need for future studies to elaborate on the theoretical underpinnings of social sustainability components.

We found no consensus on how to operationalize social sustainability, which is also indicated by others [17, 19, 50], and the same component was operationalized in divergent ways. As an example, operationalizations of social equity covered all levels in the socio-ecological model. Furthermore, the same operationalization was proposed for multiple components. For instance, neighborhood pride was suggested as an operationalization of both neighborhood quality [31], place attachment [8, 31], and social capital [12], and access to facilities was suggested as an operationalization of both social equity [3, 7, 8, 12, 13, 23, 31, 32, 51, 52, 54] and neighborhood quality [10, 50]. As social sustainability by nature is a context-dependent concept [7], the operationalizations must reflect topics of interest in the geographical region under research [10, 13], and a variety of applied operationalizations should therefore be expected. However, this diversity could further be explained by our finding that most frameworks for social sustainability, first and foremost, represent practical understandings of this concept, and researchers tend to measure social sustainability by applying concepts of interest in their specific field of research. In some studies, authors have primarily measured the physical features of a place to assess all components of social sustainability [10, 32, 51]. Furthermore, the finding that social equity was commonly operationalized as equal access to services and facilities in the local environment [8, 12, 13, 23, 31, 32, 52] clearly demonstrates this emphasis on physical features. Although unequal access to services and facilities might influence social equity, a sole focus on measuring features of the built or physical environment is somewhat narrow from a health promotion perspective. Other factors to consider when assessing social equity might be income, education, unemployment, housing affordability, or social inclusion [62, 63]. Thus, there is a need to strengthen the operationalizations of components of social sustainability by considering theoretical and empirical evidence when selecting variables for future studies. Furthermore, the social sustainability components identified in this study encompassed both the objective qualities of a place and people’s experiences of living there, and both qualitative and quantitative research are required to capture such aspects.

Within the social sustainability literature, important factors are often classified as “hard- and soft infrastructure”[8] or “physical- and non-physical factors” [7], yet, the links between these factors are quite unclear, and only three studies included relationships between different components in their frameworks [11, 50, 54]. By applying a socio-ecological perspective on social sustainability and organizing the operationalizations in a socio-ecological model, we have provided new insight into the existing literature that could be used to inform future studies. Socio-ecological models visualize the relationships between people and their environment (e.g., social, physical, and structural factors) where the individuals are represented as the center. This aligns well with definitions suggesting that social sustainability is concerned with meeting human needs, ensuring well-being, and creating places where people want to live now and in the future [3, 8, 9, 16]. Socio-ecological models are often applied to provide a simplified picture of the complexity of health determinants and are not considered causal models [64]. However, the models visualize how the levels may influence and interact with each other. Therefore, these models may guide research and practice when considering possible causal relationships in future studies and planning initiatives. For example, in a review of causal mechanisms between social relationships and health, Holt-Lunstad [65] applied a socio-ecological perspective in the summary of the results to show the relationship between social relationships and physical health, and Nutsford et al. [66] studied the relationship between access to green space and mental health using a socio-ecological approach. Our results showed that studies have primarily included aspects of the physical and social environment in frameworks for social sustainability without investigating how these factors may interact or influence population aspects. Therefore, applying socio-ecological models within future studies on social sustainability could be an essential contribution.

Another advantage of applying a socio-ecological model is that the visualization of factors and levels involved may serve as a bridge between policymakers and academics engaged in research and practice concerning social sustainability. This visualization could also make the social sustainability literature more accessible to other research disciplines, such as health promotion. From this, cross-collaboration might be established, and knowledge may be transferred. For example, in health promotion, reviews have found that policy initiatives and interventions often target individuals’ responsibility to adopt health-promoting behaviors [36, 67, 68]. In contrast, we found an emphasis on contextual factors within the social sustainability literature. Thus, the social sustainability literature may raise awareness of the importance of addressing contextual factors in research on health promotion. On the other hand, the field of health promotion may extend the social sustainability literature by providing theoretical and empirical knowledge of important factors for the health and well-being of the population.

Indeed, categorizing the identified operationalizations of social sustainability by a social-ecological model also contributed to identifying knowledge gaps in the social sustainability literature. For example, we found only five frameworks that included health and well-being in their conceptualization of social sustainability, which were operationalized using either objective measures of health, such as obesity level [3], birth rate [13], and mortality rate [13], or physical environmental measures, such as the presence of health facilities [10] or the level of air- and noise pollution [51]. First, from a health promotion point of view, these operationalizations represent a relatively narrow understanding of health and well-being, as good health is understood as a resource for everyday life where social and personal resources and physical capabilities are emphasized [25, 27]. Within this research field, health and well-being are frequently assessed by subjective measurements such as global cognitive well-being [69] and self-rated or subjective health [70]. Therefore, future studies should consider including other measurement variables when assessing health and well-being. Secondly, the conceptualization and operationalization of health and well-being in these frameworks illustrate a common need for more consideration of what the means and what the outcomes of socially sustainable development are. Because health and well-being was identified as a component of social sustainability in some frameworks [3, 10, 13, 14, 51], this component was presented alongside the others in Figure 2. However, from a health promotion point of view, health and well-being may be understood as an endpoint of social sustainability, which others have suggested [54]. Furthermore, several studies on social sustainability have assessed the means of social sustainability, such as social interaction, social capital, place attachment, and social equity [1, 8, 12, 13, 23, 31], without considering how these factors may be related to a particular outcome, such as health and well-being. Therefore, future research should elaborate and be more aware of the difference between the means and the endpoints of socially sustainable community development.

While applying a socio-ecological model to social sustainability may provide opportunities for future research and policy initiatives, some criticism and limitations have been raised in relation to these models. For example, the assumption that the levels in a socio-ecological model may interact and influence each other has not substantially been supported by theoretical or empirical studies [37, 71]. Furthermore, using a socio-ecological approach to research necessitates multilevel interventions, large-scale sampling, and cross-disciplinary collaborations, making these studies time-consuming and resource-demanding, which may operate as an obstacle [36, 71]. Nonetheless, interventions taking a socio-ecological approach would provide a more significant impact compared with a single-level approach [72].

## Strengths and limitations

This scoping review was based on a comprehensive literature search in six databases, allowing us to identify articles presenting frameworks published within different research disciplines. Hence, the components synthesized from these frameworks provide a broad picture of the conceptualization of social sustainability within the research literature. However, we only included peer-reviewed articles written in English, which could be considered a limitation. This restriction may explain the uneven distribution of identified frameworks developed for assessment purposes versus those proposed for policy planning, as frameworks for policy and planning may be more prevalent in the gray literature. While including gray literature could have provided a more balanced picture of frameworks, managing the gray literature would have been too time-consuming and burdensome at this stage, considering the complexity and immaturity of the research field. Furthermore, this study included papers published before June 2022. As the results showed that the number of articles on social sustainability has been increasing in recent years, there may be relevant studies not included in this scoping review, which represents a limitation. Throughout the review process, we adhered to the recommended guidelines for conducting a scoping review, and we strove to be transparent in every phase of the analysis, representing the key strengths of this scoping review.

## Conclusion

To conclude, the research on social sustainability is mainly conducted within research fields focusing on the physical and built environment, and hence, there is a strong tendency to operationalize social sustainability accordingly. Although several social components are considered, there seems to be limited theoretical understanding of components such as social capital, health, and well-being and how different factors interact to influence or create socially sustainable cities, neighborhoods, and local communities. Within the field of health promotion, there is a long tradition of taking a holistic and socio-ecological approach, and both theoretical and empirical knowledge on how these determinants interact to satisfy human needs and well-being exist. Therefore, the field of health promotion could add substantial knowledge and should take a more prominent role in developing the research field of social sustainability. Furthermore, knowledge from the field of social sustainability concerning the role of contextual factors in creating places for present and future generations should be adopted in health promotion research, which otherwise tends to focus on individual aspects to promote health and well-being. Cross-disciplinary collaboration has been highlighted as essential to expanding theoretical knowledge on social sustainability [29]. Finally, as only two of the identified studies were conducted in Europe, we need more research from this part of the world. Particularly interesting contexts may be the Scandinavian countries, where the welfare provisions and the minimum standard of living are relatively high in contrast to other nations [73]. Furthermore, the emphasis on participatory methods as planning tools and the importance of local communities as a unit of attention found in the Nordic countries [74] fits well within the social sustainability discourse. Therefore, more studies from Scandinavia could provide helpful insight into further conceptualizing social sustainability.

## Supplemental Material

sj-docx-1-sjp-10.1177_14034948241277863 – Supplemental material for Revisiting the conceptualization of social sustainability from a health promotion perspective: a scoping review Supplemental material, sj-docx-1-sjp-10.1177_14034948241277863 for Revisiting the conceptualization of social sustainability from a health promotion perspective: a scoping review by Ragnhild Martinsen Ånestad, Emma Charlott Andersson Nordbø and Camilla Ihlebæk in Scandinavian Journal of Public Health

sj-docx-2-sjp-10.1177_14034948241277863 – Supplemental material for Revisiting the conceptualization of social sustainability from a health promotion perspective: a scoping review Supplemental material, sj-docx-2-sjp-10.1177_14034948241277863 for Revisiting the conceptualization of social sustainability from a health promotion perspective: a scoping review by Ragnhild Martinsen Ånestad, Emma Charlott Andersson Nordbø and Camilla Ihlebæk in Scandinavian Journal of Public Health

sj-docx-3-sjp-10.1177_14034948241277863 – Supplemental material for Revisiting the conceptualization of social sustainability from a health promotion perspective: a scoping review Supplemental material, sj-docx-3-sjp-10.1177_14034948241277863 for Revisiting the conceptualization of social sustainability from a health promotion perspective: a scoping review by Ragnhild Martinsen Ånestad, Emma Charlott Andersson Nordbø and Camilla Ihlebæk in Scandinavian Journal of Public Health

sj-docx-4-sjp-10.1177_14034948241277863 – Supplemental material for Revisiting the conceptualization of social sustainability from a health promotion perspective: a scoping review Supplemental material, sj-docx-4-sjp-10.1177_14034948241277863 for Revisiting the conceptualization of social sustainability from a health promotion perspective: a scoping review by Ragnhild Martinsen Ånestad, Emma Charlott Andersson Nordbø and Camilla Ihlebæk in Scandinavian Journal of Public Health
