# Supplementary material for: Revisiting the conceptualization of social sustainability from a health promotion perspective: a scoping review
Source: Scand J Public Health. 2024 Sep 26;53(2):172–83. doi: 10.1177/14034948241277863 (PMC11907731; doi:10.1177/14034948241277863)
Supplement: sj-docx-1-sjp-10.1177_14034948241277863 – Supplemental material for Revisiting the conceptualization of social sustainability from a health promotion perspective: a scoping review [file sj-docx-1-sjp-10.1177_14034948241277863.docx]

Supplemental Material 1: Search strategy

Ovid MEDLINE(R) ALL

Search conducted 19^th^ February 2021

| Search | Query | Records retrieved |
| --- | --- | --- |
| #1 | ("socially sustainable” or “social sustainable” or “social sustainability").tw,kf. | 182 |
| #2 | “Cities”[Mesh] or "Rural population"[Mesh] | 82747 |
| #3 | (((built or rural or social or suburban or urban) adj3 environment*) or ((rural or urban or suburban) adj3 area*) or town* or city or cities or "rural population*" or "social context*" or neighborhood* or environment* or communit*).tw,kf. | 1 815 313 |
| #4 | #2 OR #3 | 1 846 506 |
| #5 | #1 AND #4 | 139 |
