# Supplementary material for: Revisiting the conceptualization of social sustainability from a health promotion perspective: a scoping review
Source: Scand J Public Health. 2024 Sep 26;53(2):172–83. doi: 10.1177/14034948241277863 (PMC11907731; doi:10.1177/14034948241277863)
Supplement: sj-docx-2-sjp-10.1177_14034948241277863 – Supplemental material for Revisiting the conceptualization of social sustainability from a health promotion perspective: a scoping review [file sj-docx-2-sjp-10.1177_14034948241277863.docx]

Supplemental Material 2: Included articles.

| Author(s) | Title | Author(s) affiliation(s) | Year | Geographic origin | Geographical scale |
| --- | --- | --- | --- | --- | --- |
| Ahmed KG | Urban Social Sustainability: A Study of the Emirati Local Communities in Al Ain. | Faculty of Engineering, Architectural Engineering Department, UAE University | 2012 | The UAE | Neighborhood |
| Akcali S and Cahantimur A | The Pentagon Model of Urban Social Sustainability: An Assessment of Sociospatial Aspects, Comparing Two Neighborhoods. | Department of Architecture, Bursa Uludag University | 2022 | Turkey | Neighborhood |
| Ali HH, Al-Betawi YN, and Al-Qudah HS | Effects of urban form on social sustainability–A case study of Irbid, Jordan. | Department of Architecture, College of Architecture & Design, Jordan University of Science & Technology  Department of Architecture, Faculty of Engineering, The Hashemite University,  Department of Architecture, Faculty of Engineering & Technology, Philadelphia University | 2019 | Jordan | Neighborhood |
| Alipour SMH and Ahmed KG | Assessing the effect of urban form on social sustainability: a proposed ‘Integrated Measuring Tools Method’ for urban neighborhoods in Dubai. | Architectural engineering department, UAE university. Amirates Center for happiness and well-being, UAE University. | 2021 | The UAE | Neighborhood |
| Ballet J, Bazin D and Mahieu FR | A policy framework for social sustainability: Social cohesion, equity and safety. | University of Bordeaux, CNRS, GREThA.  Côte d'Azur University, CNRS, GREDEG.  Fund for Research in Economic Ethics, FREE. | 2020 | Not specified | Not specified |
| Cuthill M | Strengthening the Social in Sustainable Development: Developing a Conceptual Framework for Social Sustainability in a Rapid Urban Growth Region in Australia. | UQ Boilerhouse Community Engagement Centre, University of Queensland | 2010 | Australia | Region level |
| Dempsey N, Bramley G, Power S, et.al. | The Social Dimension of Sustainable Development: Defining Urban Social Sustainability. | Oxford Institute for Sustainable Development, Oxford Brookes University. Heriot-Watt University. Scottish Executive. | 2011 | UK | Neighborhood |
| Doğu FU and Aras L | Measuring social sustainability with the developed MCSA model: Güzelyurt case. | Department of Architecture, European University of Lefke. | 2019 | Northern Cyprus | City |
| Eizenberg E and Jabareen Y | Social Sustainability: A New Conceptual Framework. | Faculty of Architecture and Town Planning, Technion, Israel Institute of Technology. | 2017 | Not specified | Not specified |
| Gomaa B and Sakr N | Social Sustainability; Maintenance of Socio-Cultural Characteristics: A Case Study of El-Raml Station. | Arab Academy for Science, Technology and Maritime Transport. | 2015 | Egypt | City |
| Hemani S, Das AK and Chowdhury A | Influence of urban forms on social sustainability: A case of Guwahati, Assam. | Aayojan School of Architecture.  Department of Design, Indian Institute of Technology (IIT).  MIT Institute of Design. | 2017 | India | City |
| Kytta M, Broberg A, Haybatollahi M, et al. | Urban happiness: context-sensitive study of the social sustainability of urban settings. | Department of Real Estate, Planning and Geoinformatics, School of Engineering, Aalto University. | 2016 | Finland | City |
| Landorf C | Evaluating social sustainability in historic urban environments. | School of Architecture, University of Queensland. | 2011 | Australia | City |
| Larimian T and Sadeghi A | Measuring urban social sustainability: Scale development and validation. | School of Architecture, Building and Civil Engineering, Loughborough University.  Aston Business School, Aston University. | 2019 | New Zealand | Neighborhood |
| Liu Y, Dijst M and Geertman S, et al. | Social sustainability in an ageing Chinese society: Towards an integrative conceptual framework | Department of Human Geography and Planning, Utrecht University.  The Center for Modern Chinese City Studies, School of Urban and Regional Science, East China Normal University. | 2017 | China | Different levels |
| Motealleh T, Zakeri S, Vakilinezhad R, et al. | Characterisation of social sustainability in a decayed urban block in Iran (a case study of: Saheb Al-Amri neighbourhood of Ghasr-Dasht, Shiraz) | Department of Architecture, Faculty of Art and Architecture, Shiraz University. | 2021 | Iran | Neighborhood |
| Murphy K | The social pillar of sustainable development: A literature review and framework for policy analysis. | School of Business and Humanities, Institute of Technology Blanchardstown. | 2012 | Not specified | Not specified |
| Opp SM | The forgotten pillar: a definition for the measurement of social sustainability in American cities. | Department of Political Science, Colorado State University. | 2017 | USA | City |
| Panda S, Chakraborty M and Misra SK | Assessment of social sustainable development in urban India by a composite index. | Piloo Mody College of Architecture.  BIT  ABIT. | 2016 | India | City |
| Shirazi MR and Keivani R | The triad of social sustainability: Defining and measuring social sustainability of urban neighbourhoods. | School of the Built Environment, Oxford Brookes University. | 2019 | Not specified | Neighborhood |
| Wang Y | The framework of social sustainability for Chinese communities: Revelation from Western Experiences. | Department of Geography and Planning, University of Liverpool | 2014 | China | Community |
| Yiftachel O and Hedgcock D | Urban social sustainability. The planning of an Australian city. | School of Architecture and Planning, Curtin University. | 1993 | Australia | City |
