# Supplementary material for: Revisiting the conceptualization of social sustainability from a health promotion perspective: a scoping review
Source: Scand J Public Health. 2024 Sep 26;53(2):172–83. doi: 10.1177/14034948241277863 (PMC11907731; doi:10.1177/14034948241277863)
Supplement: sj-docx-3-sjp-10.1177_14034948241277863 – Supplemental material for Revisiting the conceptualization of social sustainability from a health promotion perspective: a scoping review [file sj-docx-3-sjp-10.1177_14034948241277863.docx]

Supplemental Material 3: Excluded articles along with reasons for their exclusion.

| Author(s) | Title of the publication | The article does not investigate social sustainability explicitly | The article does not present a unique framework for social sustainability | The context of interest is outside the scope of this review | Theme outside the scope of this article | Wrong publication type | Framework presented in another article included in this scoping review | Not available |
| --- | --- | --- | --- | --- | --- | --- | --- | --- |
| Abramsson M and Hagberg JE | What about community sustainability? Dilemmas of ageing in shrinking semi-rural areas in Sweden. |  | x |  |  |  |  |  |
| Ahvenniemi H, Huovila A, Pinto-Seppa I, et al. | What are the differences between sustainable and smart cities? | x | x |  |  |  |  |  |
| Aksoy F and Arlı NB | Evaluation of sustainable happiness with Sustainable Development Goals: Structural equation model approach. | x | x |  |  |  |  |  |
| Amna F, Bahadir T and Ismail K | Environmental and social sustainability index (ESSI). |  | x |  |  |  |  |  |
| Aparicio C and Ramírez AE | Theoretical reflections on urban sprawl and its relationship with quality of life and social sustainability in Mexican metropolitan areas. |  | x |  |  |  |  |  |
| Asli MF and Ghamari M | Evaluation of Sahand new town based on sustainability indicators. | x | x |  |  |  |  |  |
| Bagheri M | Traces of social sustainability in garden cities Karlsruhe as a case study. |  | x |  | x |  |  |  |
| Barrado-Timon DA | The Meaning and Content of the Concept of the Social in the Scientific Discourse on Urban Social Sustainability. |  | x |  |  |  |  |  |
| Bijl R | Never Waste a Good Crisis: Towards Social Sustainable Development. |  | x |  |  |  |  |  |
| Boström M | A missing pillar? Challenges in theorizing and practicing social sustainability: Introduction to the special issue. |  | x |  |  |  |  |  |
| Botha F | The Good African Society Index. | x | x |  | x |  |  |  |
| Boyer RHW, Peterson ND, Arora P, et al. | Five Approaches to Social Sustainability and an Integrated Way Forward. |  | x |  | x |  |  |  |
| Bramley G, Dempsey N, Power S, et al. | Social sustainability and urban form: Evidence from five British cities. |  |  |  |  |  | x |  |
| Brindley T | The social dimension of the urban village: A comparison of models for sustainable urban development. |  | x |  |  |  |  |  |
| Brink M, Hengeveld GM and Tobi H | Interdisciplinary measurement: A systematic review of the case of sustainability. | x | x |  |  |  |  |  |
| Buser M and Koch C | Tales of the Suburbs? The Social Sustainability Agenda in Sweden through Literary Accounts. |  | x |  |  |  |  |  |
| Cameron L and Potvin C | Characterizing desired futures of Canadian communities. | x | x |  | x |  |  |  |
| Carpenter J | 'Social Mix' as 'Sustainability Fix'? Exploring Social Sustainability in the French Suburbs. |  | x |  |  |  |  |  |
| Cauvain J | Social sustainability as a challenge for urban scholars. |  | x |  | x |  |  |  |
| Chan P | Assessing Sustainability of the Capital and Emerging Secondary Cities of Cambodia Based on the 2018 Commune Database. | x | x |  |  |  |  |  |
| Chatzinikolaou P, Bournaris T and Manos B | Multicriteria analysis for grouping and ranking European Union rural areas based on social sustainability indicators. |  | x |  |  |  |  |  |
| Darchen S and Poitras C | Delivering social sustainability in the inner-city: the transformation of South-West Montreal, Quebec (Canada). |  |  | x | x |  |  |  |
| Davidson M | Social sustainability and the city. |  | x |  |  |  |  |  |
| De Fine Licht K and Folland A | Defining social sustainability: Towards a sustainable solution to the conceptual confusion. |  | x |  |  |  |  |  |
| Delmelle EC, Haslauer E and Prinz T | Social satisfaction, commuting and neighborhoods. |  | x |  |  |  |  |  |
| Dempsey N, Brown C and Bramley G | The key to sustainable urban development in UK cities? The influence of density on social sustainability. |  |  |  |  |  | x |  |
| Dixon T | Measuring the social sustainability of new housing development: A critical review of assessment methods. |  | x |  |  |  |  |  |
| Doyle E and Perez Alaniz M | Dichotomous impacts on social and environmental sustainability: competitiveness and development levels matter. |  | x |  |  |  |  |  |
| Dudzai C | The value of social sustainability policies to poverty reduction in Zimbabwe: A social work perspective. |  | x |  |  |  |  |  |
| Dumreicher H and Kolb B | Place as a social space: Fields of encounter relating to the local sustainability process. | x | x |  | x |  |  |  |
| Foladori G | Advances and limits of social sustainability as an evolving concept. |  | x |  |  |  |  |  |
| Ghahramanpouri A, Lamit H and Sedaghatnia S | Urban social sustainability trends in research literature. |  | x |  |  |  |  |  |
| Ghasemi E, Esfahani MN and Hosein H | A Survey on Effectiveness of Social Participating on social sustainability Dimentions (case study: Esfahan, Shahshahan Neighborhood). |  |  |  | x |  |  |  |
| Glasson J and Wood G | Urban regeneration and impact assessment for social sustainability. |  | x |  |  |  |  |  |
| Gressgård R | The power of (re)attachment in urban strategy: interrogating the framing of social sustainability in Malmo. |  | x |  | x |  |  |  |
| Grum B and Grum DK | Concepts of social sustainability based on social infrastructure and quality of life. |  | x |  |  |  |  |  |
| Gu Z and Zhang X | Framing social sustainability and justice claims in urban regeneration: A comparative analysis of two cases in Guangzhou. |  | x |  |  |  |  |  |
| Hemani S and Das AK | Humanising urban development in India: call for a more comprehensive approach to social sustainability in the urban policy and design context. |  |  |  |  |  | x |  |
| Ho WC and Cheung CK | Social sustainability for mothers in Hong Kong's low-income communities. |  | x |  |  |  |  |  |
| Holden M | Urban Policy Engagement with Social Sustainability in Metro Vancouver. |  | x |  | x |  |  |  |
| Hong S, Kweon I, Bum-Hyun L, et al. | Indicators and assessment system for sustainability of municipalities: A case study of South Korea's assessment of sustainability of cities (ASC). |  | x |  |  |  |  |  |
| Hu X, Xia B, Skitmore M, et al. | What is a sustainable retirement village? Perceptions of Australian developers. | x | x |  | x |  |  |  |
| Huang L, Yan L and Wu J | Assessing urban sustainability of Chinese megacities: 35 years after the economic reform and open-door policy. |  | x |  |  |  |  |  |
| Izadi A, Mohammadi M, Nasekhian S, et al. | Structural Functionalism, Social Sustainability and the Historic Environment: A Role for Theory in Urban Regeneration. |  |  | x | x |  |  |  |
| Jaffar N, Harun NZ and Abdullah S | The Key Determinant Factors for Social Sustainability in Traditional Settlement. |  |  |  |  | x |  |  |
| Jie H. Liangliang W and Heng CK | A social capital integrated heritage conservation approach to the regeneration of historical quarters: Toward social sustainability. |  |  |  |  |  |  | x |
| Kohon J | Interpreting the social dimension of sustainability: Connecting theory and community planning practice with a social determinants of health framework. |  | x |  |  |  |  |  |
| Kuni K | What Does Green Mean? Support holistic sustainability through thoughtful programming. |  |  |  |  | x |  |  |
| Langergaard LL | Interpreting 'the social': Exploring processes of social sustainability in Danish nonprofit housing. |  | x |  | x |  |  |  |
| Lee K and Jung H | Dynamic semantic network analysis for identifying the concept and scope of social sustainability. |  | x |  | x |  |  |  |
| Lehtonen M | The environmental-social interface of sustainable development: Capabilities, social capital, institutions. |  | x |  | x |  |  |  |
| Lin J and Yang A | Does the compact-city paradigm foster sustainability? An empirical study in Taiwan. | x | x |  | x |  |  |  |
| Liu C | Comprehensive evaluation of resource-exhausted city sustainable development: A case of Huangshi in Hubei Province. | x | x |  |  |  |  |  |
| Magee L, Scerri A and James P | Measuring Social Sustainability: A Community-Centred Approach. |  | x |  | x |  |  |  |
| Magis K | Community Resilience: An Indicator of Social Sustainability. |  | x |  | x |  |  |  |
| Martinez-Bravo M, Martinez-del-Rio J and Antolin-Lopez R | Trade-offs among urban sustainability, pollution and livability in European cities. | x | x |  | x |  |  |  |
| Medved P | Exploring the Just City principles within two European sustainable neighbourhoods. |  | x |  | x |  |  |  |
| Medved P | The essence of neighbourhood community centres (NCCs) in European sustainable neighbourhoods. | x | x |  | x |  |  |  |
| Medved P, Kim J and Ursic M | The urban social sustainability paradigm in Northeast Asia and Europe. |  | x |  |  |  |  |  |
| Mirghaderi S and Ghiri ZM | Measuring sustainable development: Linear regression approach. | x | x |  |  |  |  |  |
| Misetic A, Krnic R and Kozina G | Actors in the planning and development of Varazdin: The contribution of social sustainability research in the urban context. |  | x |  | x |  |  |  |
| Mishra N and Mishra AJ | Towards creating a socially sustainable society amid COVID- 19 pandemic: A Gandhian perspective. |  |  |  |  | x |  |  |
| Mohammed GT and Thwaites K | An Exploratory and Reflective Process of Urban Spatial Morphology within Social Sustainability: Lessons from Middle Eastern Islamic Tradition. |  | x |  | x |  |  |  |
| Mumtaz M, Khan, Chandan L, et al. | Sustainable urban development from participative planning perspective. | x | x |  |  |  |  |  |
| Neamtu B | Measuring the social sustainability of urban communities: The role of local authorities. |  | x |  | x |  |  |  |
| Neilagh ZM and Ghafourian M | Evaluation of Social Sustainability in Residential Neighborhoods. |  | x |  |  |  |  |  |
| Panda S, Chakraborty M and Misra SK | Evaluation of India's urban social sustainable development using a composite index. |  |  |  |  |  | x |  |
| Pearsall H | From brown to green? Assessing social vulnerability to environmental gentrification in New York City. |  | x |  | x |  |  |  |
| Perrons D and Dunford R | Regional development, equality and gender: Moving towards more inclusive and socially sustainable measures. |  | x |  | x |  |  |  |
| Pitarch-Garrido MD | Social Sustainability in Metropolitan Areas: Accessibility and Equity in the Case of the Metropolitan Area of Valencia (Spain). |  | x |  |  |  |  |  |
| Qian QK, Ho WKO, Ochoa JJ, et al. | Does aging-friendly enhance sustainability? Evidence from Hong Kong. | x | x |  | x |  |  |  |
| Rashidfarokhi A, Yrjänä L, Wallenius M, et al. | Social sustainability tool for assessing land use planning processes. |  |  | x | x |  |  |  |
| Rocak M, Hospers GJ and Reverda N | Searching for Social Sustainability: The Case of the Shrinking City of Heerlen, The Netherlands. |  | x |  |  |  |  |  |
| Rodrigues MMM and Franco M | Measuring the urban sustainable development in cities through a Composite Index: The case of Portugal. | x | x |  |  |  |  |  |
| Rogers DS, Duraiappah AK, Antons DC, et al. | A vision for human well-being: Transition to social sustainability. |  | x |  | x |  |  |  |
| Rogers MF | Social sustainability and the art of engagement - The small towns: Big picture experience. |  | x |  | x |  |  |  |
| Rogers SH, Gardner KH and Carlson CH | Social Capital and Walkability as Social Aspects of Sustainability. |  | x |  |  |  |  |  |
| Rogge N, Theesfeld I and Strassner C | Social Sustainability through Social Interaction-A National Survey on Community Gardens in Germany. |  |  | x | x |  |  |  |
| Santosa A, Ng N, Zetterberg L, et al. | Study Protocol: Social Capital as a Resource for the Planning and Design of Socially Sustainable and Health Promoting Neighborhoods- A Mixed Method Study. |  | x |  |  |  |  |  |
| Schlossberg M and Zimmerman A | Developing statewide indices of environmental, economic, and social sustainability: A look at Oregon and the Oregon Benchmarks. | x |  |  |  |  |  |  |
| Scott K, Park J and Cocklin C | From sustainable rural communities to social sustainability: giving voice to diversity in Mangakahia Valley, New Zealand. |  | x |  | x |  |  |  |
| Shirazi MR and Keivani R | Critical reflections on the theory and practice of social sustainability in the built environment - a meta-analysis. |  | x |  |  |  |  |  |
| Sholihah AB and Athas SIA | Analysis of Social Sustainability Models: Toward More Sustainable Indonesian Cities. |  | x |  |  |  |  |  |
| Shrivastava V and Singh J | Social sustainability of residential neighbourhood: A conceptual exploration. |  | x |  |  |  |  |  |
| Soltani S, Gu N, Ochoa JJ, et al. | The role of spatial configuration in moderating the relationship between social sustainability and urban density |  | x |  |  |  |  |  |
| Spangenberg JH and Omann I | Assessing social sustainability: Social sustainability and its multicriteria assessment in a sustainability scenario for Germany. |  | x |  |  |  |  |  |
| Taiwo MO, Samsudin S, Daud DZ, et al. | Review of the current concepts, dimensions, elements and indicators of social sustainability and social development. |  | x |  |  |  |  |  |
| Udo VE and Jansson PM | Bridging the gaps for global sustainable development: A quantitative analysis. | x | x |  |  |  |  |  |
| Ullah W, Noor S and Tariq A | The development of a basic framework for the sustainability of residential buildings in Pakistan. | x |  |  | x |  |  |  |
| Vallance SHC, Perkins HC and Dixon JE. | What is social sustainability? A clarification of concepts. |  | x |  |  |  |  |  |
| Walsh PR | Creating a values chain for sustainable development in developing nations: Where Maslow meets Porter. | x |  |  |  |  |  |  |
| Wan L and Ng E | Assessing the Sustainability of the Built Environment in Mountainous Rural Villages in Southwest China. |  | x |  | x |  |  |  |
| Wan L and Ng E | Evaluation of the social dimension of sustainability in the built environment in poor rural areas of China. |  |  | x | x |  |  |  |
| Wang J, Yan R and Zhou B | A comparative study of US and international sustainability. | x |  |  |  |  |  |  |
| Wang Y and Shaw D | The complexity of high-density neighbourhood development in China: Intensification, deregulation and social sustainability challenges. |  | x |  |  |  |  |  |
| Wei Z, Wang B, Chen T, et al. | Community development in urban Guangzhou since 1980: A social sustainability perspective. |  | x |  |  |  |  |  |
| Weingaertner C and Moberg A | Exploring Social Sustainability: Learning from Perspectives on Urban Development and Companies and Products. |  | x |  |  |  |  |  |
| Winterton R, Butt A, Jorgensen B, et al. | Local government perspectives on rural retirement migration and social sustainability. |  | x |  | x |  |  |  |
| Wolsko C, Marino E, Doherty TJ, et al. | Systems of access: A multidisciplinary strategy for assessing the social dimensions of sustainability. |  | x |  | x |  |  |  |
| Wu G, Duan K, Zuo J, et al. | Integrated Sustainability Assessment of Public Rental Housing Community Based on a Hybrid Method of AHP-Entropy Weight and Cloud Model. | x | x |  |  |  |  |  |
| Yi P, Dong Q and Li W | Evaluation of city sustainability using the deviation maximization method. | x | x |  |  |  |  |  |
| Yi P, Li W and Zhang D | Assessment of city sustainability using MCDM with interdependent criteria weight. | x | x |  |  |  |  |  |
| Zhang Q, Yung EHK and Chan EHW | Comparison of perceived sustainability among different neighbourhoods in transitional China: The case of Chengdu. | x |  |  |  |  |  |  |
| Zhen L, Cao S, Wei Y, et al. | Comparison of sustainability issues in two sensitive areas of China. | x |  |  |  |  |  |  |
| Ziaesaeidi P and Cushing DF | The social sustainability of neighbourhood-schools: a qualitative study with Iranian children and youth about their neighbourhood perceptions. |  |  | x | x |  |  |  |
| Zou T, Su Y and Wang Y | Examining relationships between social capital, emotion experience and life satisfaction for sustainable community. |  | x |  | x |  |  |  |
