# Supplementary material for: Revisiting the conceptualization of social sustainability from a health promotion perspective: a scoping review
Source: Scand J Public Health. 2024 Sep 26;53(2):172–83. doi: 10.1177/14034948241277863 (PMC11907731; doi:10.1177/14034948241277863)
Supplement: sj-docx-4-sjp-10.1177_14034948241277863 – Supplemental material for Revisiting the conceptualization of social sustainability from a health promotion perspective: a scoping review [file sj-docx-4-sjp-10.1177_14034948241277863.docx]

Supplemental Material 4: Data extraction tool

| Type of information | Data to extract |
| --- | --- |
| Information concerning the publication | Title of the publication |
|  | Author(s) |
|  | Author(s) affiliation(s) |
|  | Year published |
|  | Name of journal/publisher |
|  | Type of literature (Journal article, book section, etc.) |
|  | Study type |
|  | Methods |
|  | Main aim/objective of the study |
|  | Country of interest in the publication |
|  | Geographical scale (neighborhood/local community/city) |
| Information concerning the framework | Definition or description of social sustainability |
|  | Description of the framework |
|  | Description of main concepts |
|  | Description of operationalization |
